# Functional Inversion of Trisodium Citrate from a [0001] Growth Suppressor to a Promoter in ZnO Thin Film Fabrication

**DOI:** 10.1021/acsomega.5c07099

**Published:** 2026-01-09

**Authors:** Lo Tuan Son, Yuta Kubota, Hajime Wagata, Nobuhiro Matsushita

**Affiliations:** † Department of Materials Science and Engineering, School of Materials and Chemical Technology, 13290 Institute of Science Tokyo, 2-12-1 Ookayama, Meguro, Tokyo 152-8550, Japan; ‡ Department of Applied Chemistry, 83923 School of Science and Technology, Meiji University, 1 Chome-1-1 Higashimita, Tama Ward, Kawasaki, Kanagawa 214-8571, Japan

## Abstract

Trisodium citrate is a well-established molecular modifier for ZnO thin films renowned for its role in suppressing [0001] growth by selectively passivating the (0002) facet in conventional, slow-growth fabrication methods. A paradoxical functional inversion is demonstrated herein: under fast-growth conditions using a mist-based fabrication, trisodium citrate acts as a powerful promoter, yielding well-aligned, high-aspect-ratio nanorods with a strong [0001] orientation. This functional switch is attributed to a fundamental change in the dominant growth unit, from the neutral Zn­(OH)_2_ species prevalent under slow-growth conditions to the anionic Zn­(OH)_4_^2–^ complex enabled by the fast-growth process. This change in growth unit chemistry activates the transformation of citrate from a static suppressor into a mobile promoter via surface diffusion and lowering the crystallization energy barrier. These findings establish a new framework where the synthesis kinetics dictate the dominant growth unit chemistry, which, in turn, governs the ultimate function of a molecular additive, offering a new strategy for the rational fabrication of anisotropic nanostructures.

## Graphical abstract

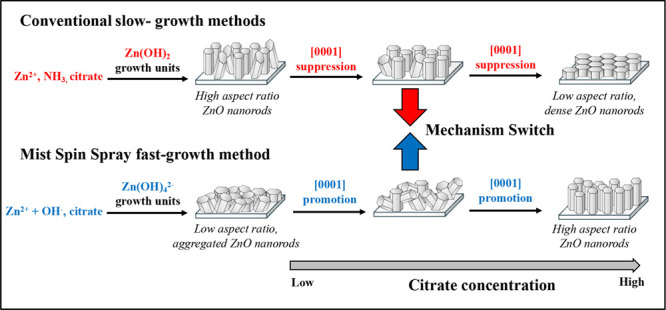

## 1 Introduction

The controlled fabrication of zinc oxide (ZnO) thin films with tailored nanostructures is of significant interest for a wide range of applications.^1–4^ A key strategy to manipulate the morphology of ZnO thin films is through the use of molecular additives, with trisodium citrate (citrate) being a canonical example. A vast body of literature has established that citrate functions as a highly specific [0001] growth suppressor for ZnO thin films.^2,5–8^ This effect is widely understood to originate from the selective adsorption of carboxyl groups of citrate onto the positively charged, Zn-terminated (0002) crystal facet, which passivates the surface and consequently inhibits growth along the [0001] direction.^2,6,7,9^ The specificity of this interaction has been extensively confirmed not only through numerous experimental reports but also by theoretical simulations.^10,11^

To achieve ZnO thin films with high homogeneity and strong substrate adhesion, the solution-based fabrication often relies on “slow-growth methods,” which are based on the controlled reaction between a zinc salt and a weak base, proceeding through complexes or hydroxide intermediates.^12^ Approaches such as chemical bath deposition,^6^ spin spray,^13,14^ and hydrothermal^15^ represent classic examples that operate on this principle. The conventional focus on these slow, controlled processes may explain why phenomena that are unique to different kinetic regimes have been largely overlooked. In stark contrast to these slow processes are “fast-growth methods,” characterized by significantly accelerated reaction rates. The mist spin spray (MSS) technique is one such unique method that operates in a highly alkaline aqueous environment. It uniquely overcomes the challenge of bulk precipitation, typically associated with fast reactions, by precisely controlling and limiting the volume of reacting mist particles on the substrate at any given moment, thus ensuring a uniform and adherent thin film. This method has been previously established for fabricating and investigating CuO,^16^ Cu_2_O,^17^ and ZnO thin films.^18^

This stark difference in kinetic regimes raises a critical question: can the function of a well-established growth suppressor be inverted, transforming it into a promoter, simply by switching from a slow- to a fast-growth method? In this work, we demonstrate a paradoxical reversal of citrate under the fast-kinetic conditions of MSS fabrication. We show that citrate transforms from a suppressor into a powerful promoter of [0001] growth, yielding well-aligned, high-aspect-ratio ZnO nanorods. While a few reports on other fast-growth methods have hinted at a similar promoter effect, the underlying mechanisms have remained poorly understood.^19,20^ Through a detailed investigation of the nanostructure, surface, and optical properties, we elucidated the chemical mechanisms governing this functional switch. We attribute the reversal to the interplay between the dominant Zn­(OH)_4_^2–^ growth unit and a unique surface dynamic and the lowering of the crystallization energy barrier. We further explore the consequence of these changes on their photocatalytic performance. These findings provide a new framework for kinetically controlling crystal habits, demonstrating that the function of a molecular additive can be fundamentally dictated by the dominant growth unit chemistry.

## 2 Materials and Methods

### 2.1 Thin Films Fabrication

ZnO thin films were fabricated using a MSS apparatus, with the experimental setup detailed in Figure S1 and based on our previous work.^16–18^ All chemical reagents were purchased from Fujifilm Wako Pure Chemical and used as received. Prior to deposition, glass substrates (40 × 30 × 0.17–0.25 mm) were treated with UV/ozone for 10 min to render the surface hydrophilic. The film growth was initiated by the reaction of a source solution (0.075 M zinc nitrate, Zn­(NO_3_)_2_, 99%) and a reaction solution. The reaction solution contained sodium hydroxide (NaOH, 97%) at varying concentrations (0.15, 0.225, or 0.3 M) to achieve Zn^2+^/OH^–^ molar ratios of 1:2, 1:3, and 1:4. For select experiments, additives were introduced into the reaction solution, including trisodium citrate (95%, 0.02–0.1 M) or other carboxylic acids (glycolic, malic, maleic, and succinic; 95–97%) at a fixed concentration of 0.06 M. For some control experiments, ammonia (NH_3_) was added to the reaction solution to achieve a final volume concentration of 10–30%. Deposition was performed at a substrate temperature of 80 °C for durations of 5, 10, or 30 min. Key process parameters were held constant: a table rotation speed of 150 rpm, a carrier gas flow rate of 8 L/min per nozzle, and a nozzle-to-substrate distance of 3 mm. Following deposition, the films were cleaned by sonication in deionized water for 10 min and subsequently air-dried. The sample notations and corresponding fabrication conditions are summarized in Table 1.

**Table 1:** Denotation and Condition of Fabricated ZnO Thin Films

		reaction solution		
sample name	source solution	base	additives	fabrication time
ZnO_C*x*_	0.075 M Zn(NO_3_)_2_	0.3 M NaOH (molar ratio Zn^2+^/OH^–^ of 1:4)	*x* M citrate (*x* = 0, 0.02, 0.04, 0.06, and 0.1)	30 min
ZnO_C*x*T*y*_		0.3 M NaOH (molar ratio Zn^2+^/OH^–^ of 1:4)	*x* M citrate (*x* = 0, 0.02, and 0.06)	*y* min (*y* = 5, 10)
ZnO_citrate_/ZnO_glycolic_/ZnO_malic_/ZnO_maleic_/ZnO_succinic_		0.3 M NaOH (molar ratio 1:4)	0.06 M citrate/glycolic/malic/maleic/succinic acid	30 min
ZnO_C*x*R1:*z*_		0.15 and 0.225 M NaOH (molar ratio Zn^2+^/OH^–^ of 1:*z*, *z* = 2 and 3)	*x* M citrate (*x* = 0 and 0.06 M)	30 min

### 2.2 Characterization

The surface morphology and cross sections of the ZnO thin films were examined by using scanning electron microscopy (SEM, S-5500, Hitachi). Nanostructure size distributions were directly analyzed from the SEM images. The crystal structure of the films was characterized through X-ray diffraction (XRD) measurements in θ–2θ mode, covering a 2θ range from 20 to 70°, with a scan rate of 5°/min and a resolution of 0.01° using an X-ray diffractometer (MiniFlex600, Rigaku). Fourier transform infrared (FT-IR) spectroscopy (IRPrestige-21, Shimadzu), in the wavenumber range of 4000–400 cm^–1^, was used to confirm the removal of organic or process-related residues after cleaning. Optical properties were evaluated through UV–VIS spectroscopy using a spectrophotometer (V-670, JASCO) in the wavelength range of 320 to 480 nm, with a scan rate of 40 nm/min and a resolution of 0.2 nm. Elemental composition and electronic states were determined via X-ray photoelectron spectroscopy (XPS) using a spectrometer (JPS-9010). For survey scans, an Al K_α_ X-ray source was used with a pass energy of 50 eV. The optical emission properties were studied by using photoluminescence (PL) spectroscopy on a fluorescence spectrophotometer (F-7100, Hitachi). The PL spectra were recorded from 350 to 650 nm with an excitation wavelength of 339 nm. The scan rate was set to 240 nm/min with a resolution of 0.2 nm.

### 2.3 Photocatalyst Activity

ZnO_C*x*_ (*x* = 0–0.1 M) thin films with dimensions of 2 × 3 cm were immersed in Petri dishes containing 40 mL of a 2 ppm methylene blue (MB) solution. The UV source utilized comprised four Panasonic GL8 UV lamps (2.5 W each), with a primary emission wavelength of 254 nm. Before the photocatalytic test, the samples were kept in the dark for 1 h to achieve equilibrium. Subsequently, 2 mL of the solution was extracted, and UV–VIS spectroscopy was employed to measure the absorbance of MB at a wavelength of 665 nm after each 1 h. The duration of the MB decomposition reaction was set to 6 h. The efficiency (*H*) of MB decomposition was calculated using the formula: *H* = (*A*_0_ – *A*_*t*_)/*A*_0_, where *A*_0_ and *A_t_* represent the absorbance of the MB solution at the initial time and at time *t*, respectively. The rate constant (*k*) for the reaction was calibrated under the assumption of first-order kinetics, following the equation *kt* = ln­(*A*_0_/*A*_*t*_)*,* where *t* denotes the reaction time.

## 3 Results and Discussion

### 3.1 Effects of Citrate on the Physicochemical Properties of ZnO Films

#### 3.1.1 Films Appearance and Surface Purity Analysis

All fabricated films appeared as opaque, white, and homogeneous layers with strong adhesion to the glass substrate after sonication, as shown in Figure S2. Film purity and the nature of the additive interaction were assessed using FT-IR spectroscopy (Figure 1). The ZnO_C0.06_ thin films before sonicating displayed strong, broad peaks characteristic of citrate (around 1350, 1560, and 3300 cm^–1^)^9,20,21^ as well as weaker, sharp peaks at 830 and 1790 cm^–1^ attributed to sodium nitrate byproduct,^22^ which is soluble and can be easily removed during the cleaning step. Critically, all of the citrate peaks vanished completely after the ultrasonic cleaning step, rendering their FT-IR spectrum identical with that of the bare glass substrate. This confirms that citrate was only a loose, surface-bound species that is easily removed. This finding is in sharp contrast to conventional slow-growth methods, where citrate is often incorporated as a stable impurity within the ZnO crystal lattice.^6,20,23^ The fact that citrate acts as a transient surface agent rather than as a permanent impurity is a crucial piece of evidence. It suggests a fundamentally different, more dynamic surface interaction mechanism under fast-kinetic conditions compared to the static inhibition and entrapment seen in slow-growth regimes.

**Figure 1:**
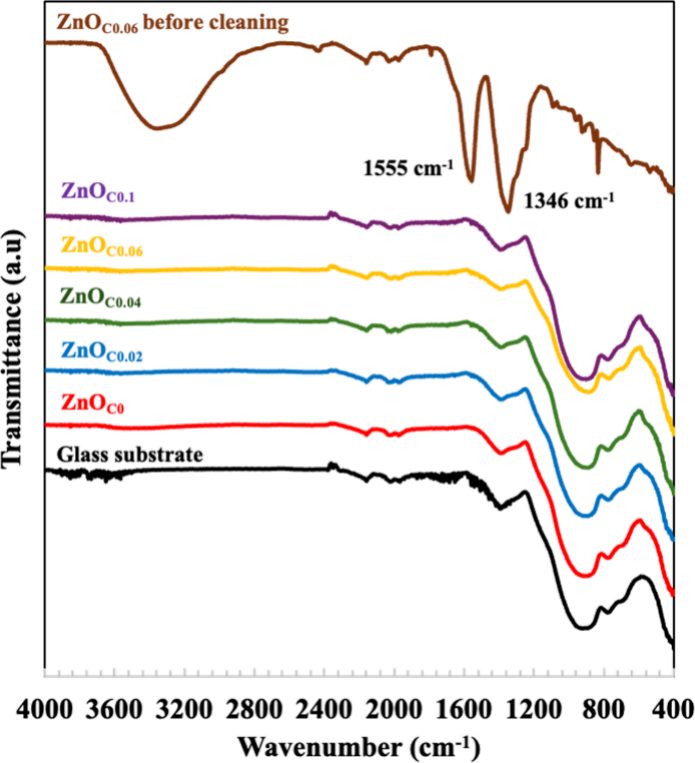
FT-IR spectra of ZnO_C*x*_ thin films (*x* = 0–0.1).

#### 3.1.2 Citrate-Induced Anisotropic Growth: A Morphological and Structural Analysis

All fabricated thin films exhibited excellent homogeneity and were crack-free over a large area, as confirmed by low-magnification SEM images (Figure S3). High-magnification SEM images (Figure 2) revealed a dramatic evolution in the ZnO nanostructure. The ZnO_C0_ thin film consisted of highly agglomerated, randomly oriented nanorods, while the addition of citrate promoted the formation of dense arrays of well-separated, vertically aligned nanorods. This promotional effect was most directly evidenced by their cross-sectional SEM images, which showed a remarkable increase in the overall film thickness from 123 nm in ZnO_C0_ to 750 nm in ZnO_C0.1_ thin films. This more than 6-fold increase in thickness provided definitive proof that citrate acted as a powerful promoter for growth along the vertical [0001] direction. This improved ordering can be attributed to the suppression of secondary nucleation in the early stages of growth, as suggested by a time-evolution investigation (Figure S4). The anisotropic nature of this promotion was clearly quantified by the data presented in Figure 3. While the average nanorod diameter remained relatively stable (from 62.7 to 65.0 nm), the average length increased dramatically from 136.7 nm in ZnO_C0_ to 566.3 nm in ZnO_C0.1_ thin films. Consequently, the nanorod aspect ratio was significantly enhanced from 2.18 to 8.71. This profound transformation in physical morphology was a direct consequence of an underlying change in their crystallographic properties, as confirmed by the XRD analysis (Figure 4). The XRD patterns showed a systematic enhancement of the (0002) peak intensity, perfectly corroborating the vertical alignment observed in the SEM images. This preferred *c*-axis orientation was also evident at the crystallite level (Table 2). The crystallite size calculated for the [0001] direction more than doubled, growing from 33.8 nm in ZnO_C0_ to 70.9 nm in ZnO_C0.1_ thin films, while the sizes along other crystallographic directions remained largely unchanged.

**Figure 2:**
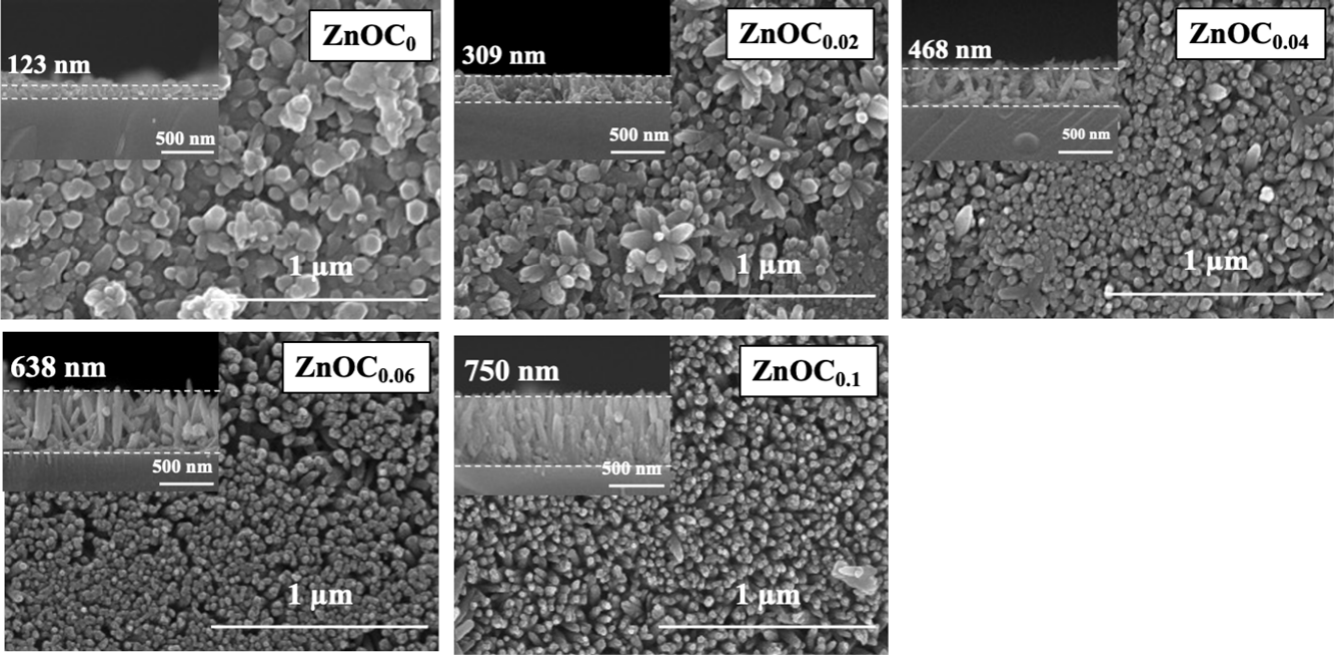
SEM images (50,000 times magnification and cross-section) of ZnO_C*x*_ thin films (*x* = 0–0.1).

**Figure 3:**
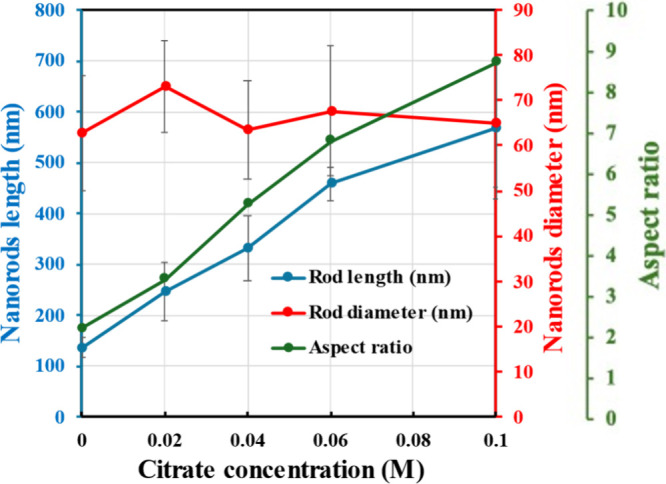
Length, diameter, and aspect ratio of ZnO nanorods in ZnOC_*x*_ thin films (*x* = 0–0.1) as a function of citrate concentration, quantified from cross-section SEM images.

**Figure 4:**
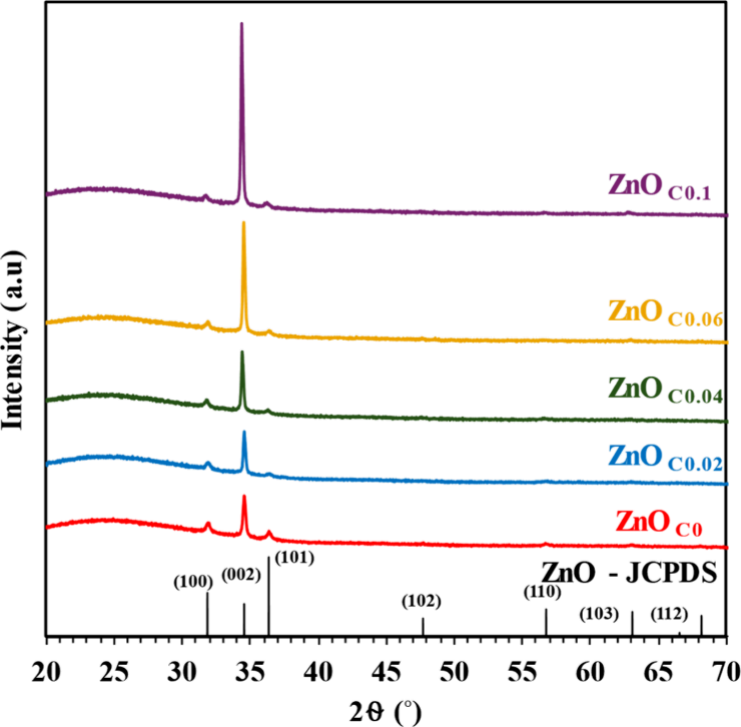
XRD spectra of ZnO_C*x*_ thin films (*x* = 0–0.1).

**Table 2:** XRD Profiles of ZnO_C*x*_ Thin Films (*x* = 0, 0.02, 0.04, 0.06, and 0.1)

	peak position (°)			crystal constant (nm)			crystallite size (nm)		
sample	(101̅0)	(0002)	(101̅1)	(101̅0)	(0002)	(101̅1)	[101̅0]	(0002)	[101̅1]
ZnO_C0_	31.73	34.39	36.23	0.2818	0.2606	0.2478	18.3968	33.8030	19.2800
ZnO_C0.02_	31.76	34.39	36.23	0.2816	0.2606	0.2477	20.5868	44.1626	20.7163
ZnO_C0.04_	31.68	34.36	36.17	0.2822	0.2608	0.2481	21.4780	52.6645	22.3316
ZnO_C0.06_	31.80	34.48	36.28	0.2812	0.2599	0.2474	23.7673	54.3872	25.7587
ZnO_C0.1_	31.71	34.36	36.20	0.2820	0.2608	0.2479	24.8585	70.8585	29.4175
ZnO-JCPDS	31.85	34.55	36.36	0.2807	0.2594	0.2469			

#### 3.1.3 Surface Chemistry, Electronic Structure, and Optical Properties

The citrate-induced structural modifications were accompanied by profound changes in the surface chemistry and electronic properties of the ZnO thin films. Analysis of the XPS data provided critical insights. The O 1s spectra (Figure 5a and Table 3) were deconvoluted into three components: lattice oxygen (O_l_), oxygen vacancies (O_v_), and surface hydroxyl groups (O_h_).^24,25^ Two key findings emerged: first, the O_h_ peak at ∼532.4 eV was completely absent in films fabricated with higher citrate concentrations (≥0.04 M), providing direct evidence that citrate displaced native hydroxyl groups on the growth surface. Second, the relative intensity of the O_v_ peak systematically increased with citrate, indicating the formation of a more defect-rich state consistent with the fast-growth conditions. Furthermore, analysis of the Zn 2p spectra (Figure 5b) revealed a slight positive shift in binding energies upon citrate addition, reflecting changes in the Zn^2+^ chemical environment due to the increase in neighboring oxygen vacancies.^24^ This was complemented by the increase in the surface Zn/O_l_ ratio from a near-stoichiometric 0.96 to over 2.1 when citrate was added, a trend in excellent agreement with the enhanced [0001] orientation, which exposes more of the Zn-terminated (0002) facet. These changes in the surface and defect structure were directly reflected in the optical emission properties. The unnormalized PL spectra (Figure 6a) of ZnO_C*x*_ thin films revealed a pronounced quenching effect, where the absolute PL intensity, particularly the near-band-edge (NBE) emission, decreased significantly as the citrate concentration increased. This occurred even though the films became substantially thicker, indicating that the fast, kinetically driven growth process introduces nonradiative recombination centers.^26^ Furthermore, analysis of the spectra in Figure 6b, which were normalized to the defect band emission peak, showed that the intensity of the NBE emission relative to the defect band increased with citrate, suggesting an improvement in the structural order within the crystalline domains, which aligns perfectly with the XRD results. Crucially, the relative contribution of the green emission band (∼550 nm), characteristic of singly ionized oxygen vacancies, increased dramatically, providing compelling optical confirmation of the higher vacancy concentration observed in XPS. Ultimately, these modifications impacted the bulk optical properties of the films. The UV–vis spectra (Figure 7a) showed a nonmonotonic trend in transmittance; it initially increased for the ZnO_C0.02_ thin films due to reduced nanostructure agglomeration, but then decreased as the film thickness became dominant at higher citrate concentrations.^27^ Additionally, a distinct red-shift in the absorption edge was observed with the absorption peak shifting from 342.8 nm in ZnO_C0_ to 348.6 nm in ZnO_C0.06_ thin films. This corresponds to a decrease in the optical band gap from 3.355 to 3.290 eV (Figure 7b). This band gap narrowing is attributed to the synergistic effect of improved crystallinity and the increased density of oxygen vacancy states, which are known to introduce energy sublevels just below the conduction band.^28,29^

**Figure 5:**
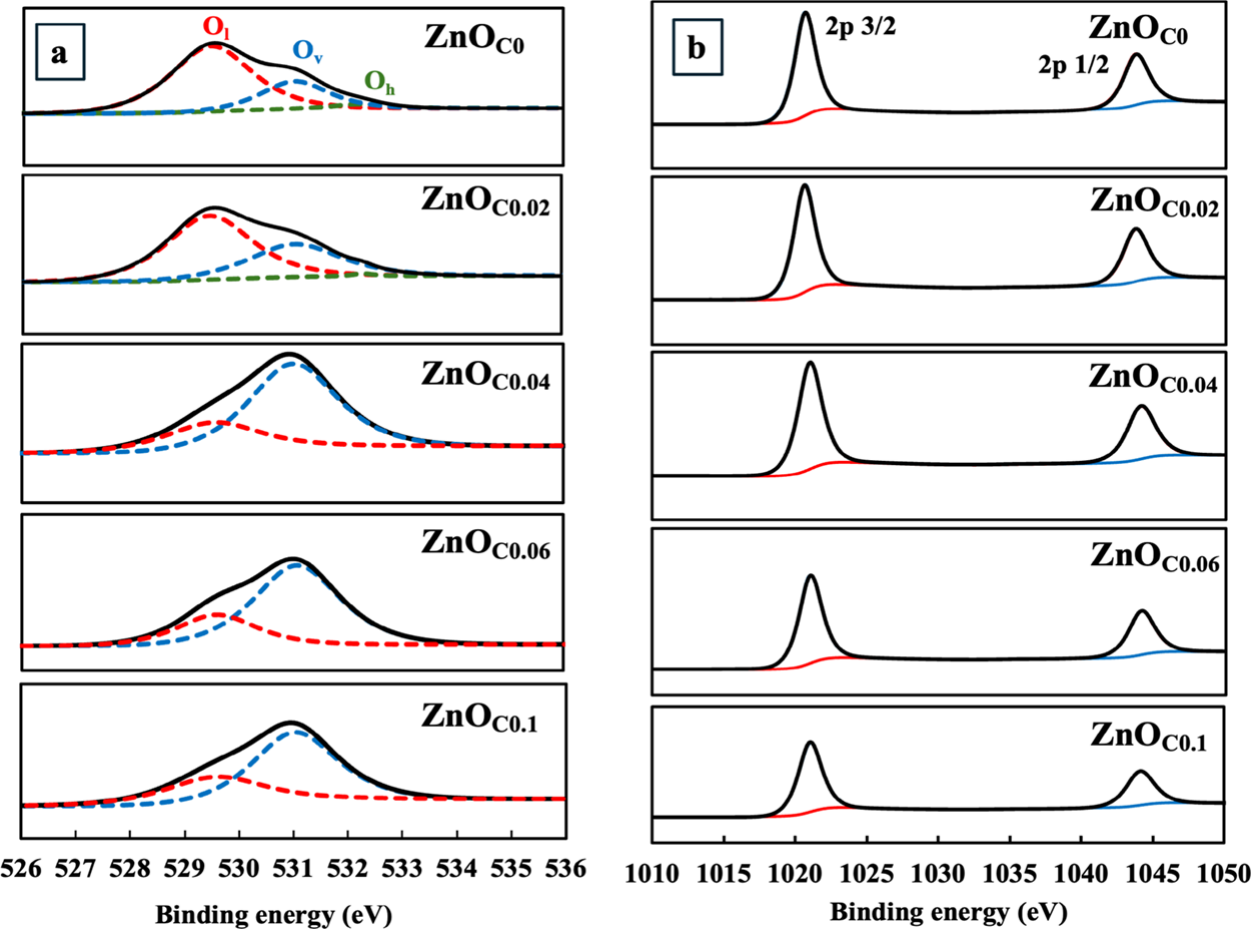
(a) O 1s and (b) Zn 2p XPS spectra of ZnO_C*x*_ thin films (*x* = 0–0.1).

**Figure 6:**
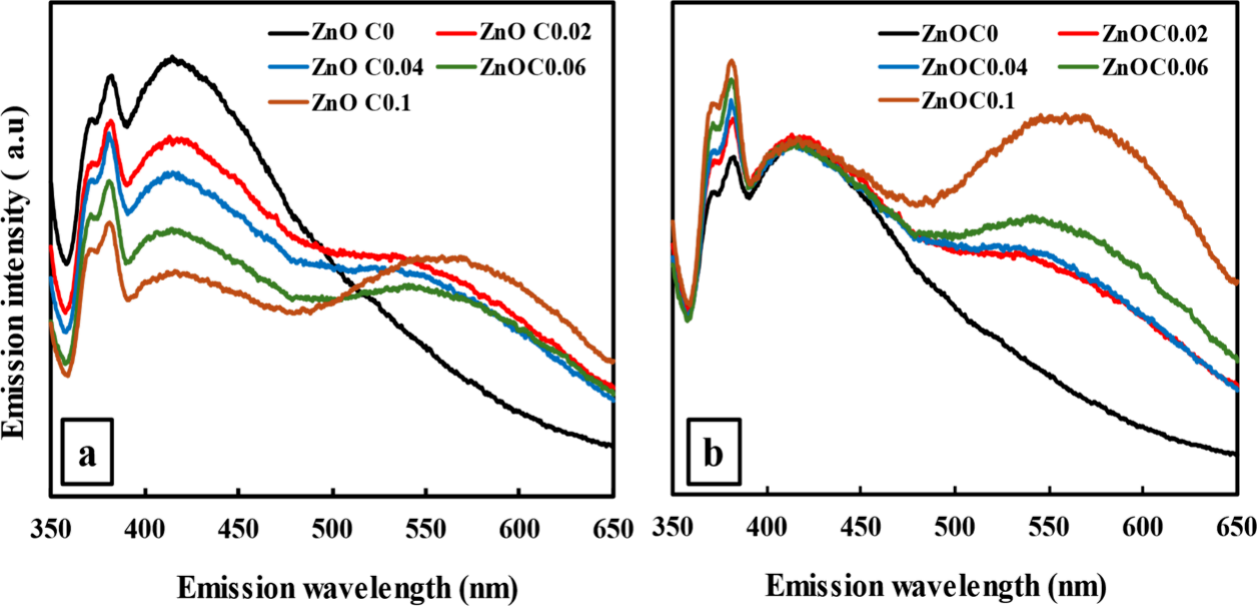
(a) Unnormalized and (b) normalized PL spectra of ZnO_C*x*_ thin films (*x* = 0–0.1).

**Figure 7:**
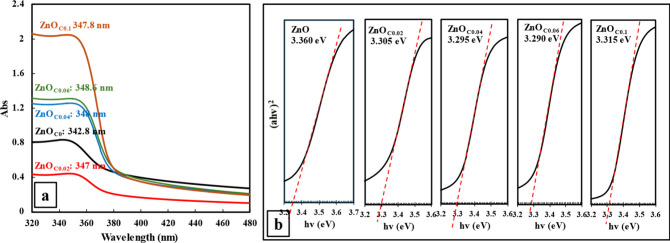
(a) UV–vis absorbance spectra, showing absorption peak positions, and (b) Tauc plots, showing band gap values of ZnO_C*x*_ thin films (*x* = 0–0.1).

**Table 3:** XPS Peak Position and Zn/O Ratio of ZnO_C*x*_ Thin Films (*x* = 0, 0.02, 0.04, 0.06, and 0.1)

	Zn 2p		O 1s				
sample	2p_3/2_	2p_1/2_	O_l_	O_v_	O_h_	Zn/O_l_	Zn/O_v_
ZnO_C0_	1043.71	1020.65	529.48	531.02	532.24	0.958	2.773
ZnO_C0.02_	1043.71	1020.65	529.46	531.04	532.31	0.980	1.832
ZnO_C0.04_	1044.08	1021.01	529.55	531.00	N/A	2.129	0.802
ZnO_C0.06_	1044.12	1021.07	529.60	531.07	N/A	1.722	0.625
ZnO_C0.1_	1044.13	1021.06	529.58	531.01	N/A	1.664	0.797

#### 3.1.4 Photocatalytic Activities

Figure 8a,b presents the photocatalytic performance of ZnO thin films in the MB decomposition reaction. ZnO_C0_ thin films exhibited a degradation efficiency of 81.23% after 6 h, which increased to 94.35% for the ZnO_C0.1_ thin films. Kinetic analysis, assuming a pseudo-first-order reaction, revealed that the reaction rate constant increased as citrate concentration increased. The rate constant for ZnO_C0.1_ thin films was 0.4835 h^–1^, 1.83 times higher than that for ZnO_C0_ thin films, 0.2648 h^–1^. The improved photocatalytic performance can be attributed to two main key factors. First, morphological changes induced by citrate, as SEM results showed, a transformation from low-aspect-ratio, aggregating nanorods to more separated, high-aspect-ratio nanorods. This morphological alteration increased the surface area of the ZnO thin films, which enhanced their photocatalytic properties. Additionally, XPS and PL analyses revealed that citrate increased the oxygen vacancy concentration in the ZnO thin films. Numerous studies have demonstrated the critical role of oxygen vacancies in enhancing the photocatalytic activity of ZnO.^29–32^ Therefore, the combination of morphological and electronic structural changes resulted in a significantly improved photocatalytic performance for citrate-treated ZnO thin films.

**Figure 8:**
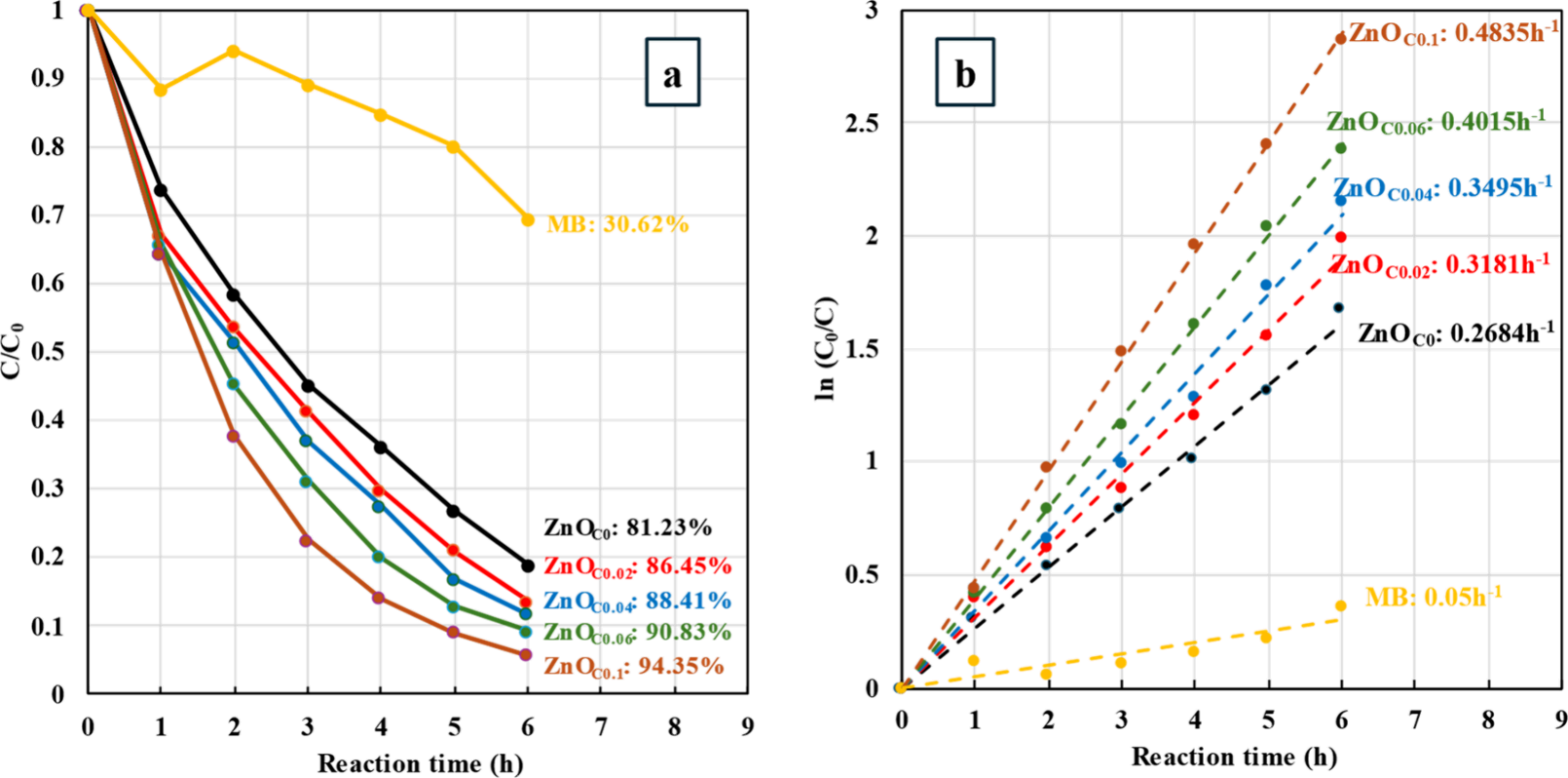
(a) MB degradation efficiency and (b) pseudo-first-order kinetic analysis of MB degradation for ZnO_C*x*_ thin films (*x* = 0–0.1).

### 3.2 Experimental Validation of the Growth Unit Hypothesis

#### 3.2.1 Identifying the Dominant Growth Unit: Effects of Molar Ratio and Ammonia

To experimentally validate the opposing effects of citrate on different ZnO growth units, we manipulated the fabrication pathway in two distinct ways: first by varying the Zn^2+^/OH^–^ molar ratio and second by introducing ammonia (NH_3_) to simulate slow-growth conditions. While the speciation of zinc is fundamentally governed by pH,^33,34^ its direct in situ measurement within the dynamic mist on the hot substrate is not feasible. Therefore, consistent with our previous work,^18^ the Zn^2+^/OH^–^ molar ratio is employed as a robust proxy to control the chemical environment and thus dictate the dominant growth unit. This principle is described by the following general reactions:

$${\mathrm{Zn}}^{2+}+x{\mathrm{OH}}^{-}\to \mathrm{Zn}(\mathrm{OH}{{)}_{x}}^{(x-2)-}$$ 1

$$\mathrm{Zn}(\mathrm{OH}{{)}_{x}}^{(2-x)-}\to \mathrm{ZnO}+(x-2){\mathrm{OH}}^{-}+H_{2}O$$ 2

where *x* = 2 or 4 corresponds to Zn­(OH)_2_ and Zn­(OH)_4_^2–^, respectively. The SEM results in Figure S6 illustrate this effect. At a molar ratio of 1:2, where Zn­(OH)_2_ is the primary growth unit, the ZnO_C0R1:2_ thin films consist of small nanoparticles (∼30 nm). The addition of citrate only causes these nanoparticles to become slightly larger and more rod-like (∼70 nm) but fails to produce vertically aligned nanorods. Similarly, at a 1:3 ratio, where the growth units consist both of Zn­(OH)_2_ and Zn­(OH)_4_^2–^, citrate addition merely transforms a hybrid nanoparticle/nanorod structure (ZnO_C0R1:3_ thin films) into more defined flower-like morphologies (ZnO_C0.06R1:3_). This demonstrates that when Zn­(OH)_2_ is a major component of the growth pathway, the [0001] growth promotion is severely limited, starkly contrasting its role at the 1:4 ratio where Zn­(OH)_4_^2–^ dominates.

In a second approach, we simulated conventional slow-growth conditions by adding NH_3_ (10–30%) into our standard fast-growth system (0.06 M citrate, Zn^2+^/OH^–^ molar ratio of 1:4). The resulting morphology is shown in Figure 9. The addition of NH_3_ led to nanorods with an increased diameter but a lower number density. This indicates that [0001] growth was partially inhibited while [10 1̅ 0] growth was relatively promoted compared to the thin films without NH_3_. This occurs because NH_3_ sequesters Zn^2+^ into the stable Zn­(NH_3_)_4_^2+^ complex. Although this complex is an intermediate, it ultimately generates Zn­(OH)_2_ as the final growth unit, following the well-established pathway in conventional slow-growth methods:

$${\mathrm{NH}}_{3}+H_{2}O⇌{{\mathrm{NH}}_{4}}^{+}+{\mathrm{OH}}^{-}$$ 3

$${\mathrm{Zn}}^{2+}+4{\mathrm{NH}}_{3}⇌\mathrm{Zn}({\mathrm{NH}}_{3}{{)}_{4}}^{2+}$$ 4

$$\mathrm{Zn}({\mathrm{NH}}_{3}{{)}_{4}}^{2+}+x{\mathrm{OH}}^{-}⇌\mathrm{Zn}(\mathrm{OH}{{)}_{4-x}}^{(x-2)-}+4{\mathrm{NH}}_{3}$$ 5

where the formation of the Zn­(NH_3_)_4_^2+^ complex and the weak basicity of NH_3_ ensure that *x* = 2, and Zn­(OH)_2_ is the prevalent growth unit. Taken together, these two distinct experimental approaches converge on a single unambiguous conclusion, which is visually summarized in Figure 10. Whether the Zn­(OH)_2_ growth unit is generated via a low Zn^2+^/OH^–^ molar ratio or via the introduction of an ammonia complexing agent, its presence consistently leads to the suppression of [0001] growth by citrate. Conversely, the powerful growth promotion of citrate is exclusively linked to the presence of a Zn­(OH)_4_^2–^ growth unit. This provides definitive experimental proof that the function of citrate is fundamentally dictated by the chemical identity of the growth unit it interacts with.

**Figure 9:**
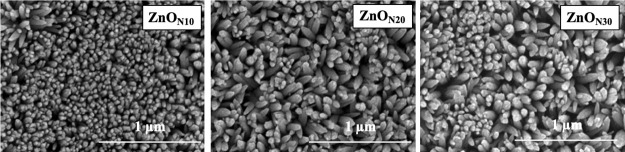
SEM images (50,000 times magnification) of ZnO_N*t*_ thin films (*t* = 10, 20, 30).

**Figure 10:**
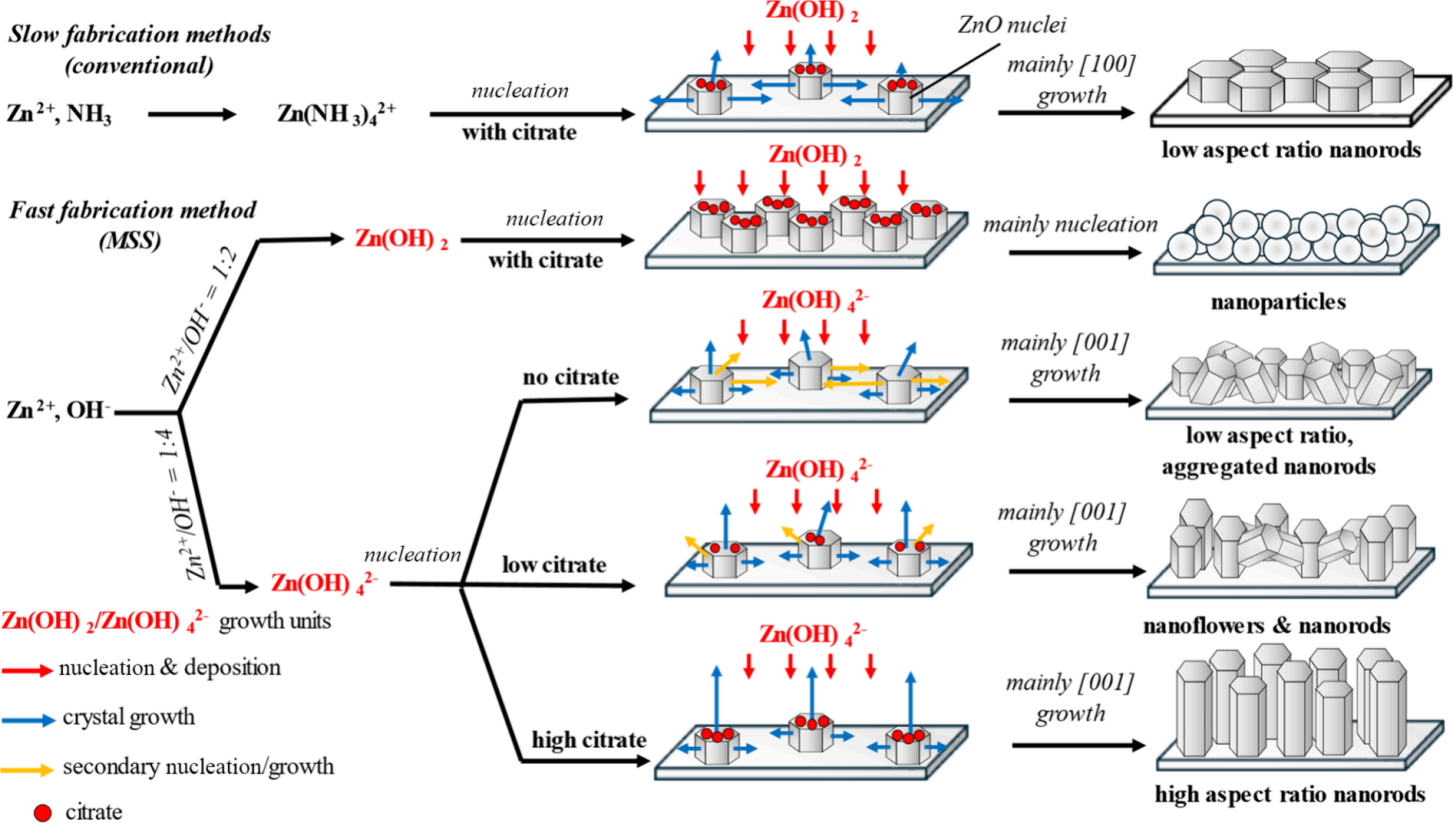
Summary of the trend in nucleation and crystal growth of ZnO thin films fabricated in various methods and conditions.

#### 3.2.2 Specificity and Dynamics of the Citrate-ZnO Interaction

Having established that the Zn­(OH)_4_^2–^ growth unit is a prerequisite for the promotional effect, the next logical question is whether this effect is universal to any surface modifier or whether it requires a specific molecular interaction. To investigate this, a series of other carboxylic acids, glycolic, malic, maleic, and succinic acid were tested as alternatives to citrate under the same Zn­(OH)_4_^2–^-rich conditions (molar ratio Zn^2+^/OH^–^ of 1:4). The resulting morphologies and crystal structures, shown in Figures 11 and S5 respectively, reveal that these additives had only a minimal to modest impact. None were able to induce the dramatic, highly oriented [0001] growth observed with citrate, indicating that the identity of the molecular additive is indeed critical. We propose that the reason for this distinction lies in their varying adsorption strengths on the ZnO surface. This was quantified by FT-IR spectroscopy (Figure 12) by measuring the peak shifts of the carboxylate (−COO^–^) groups upon interaction with ZnO. The FT-IR spectrum of the unwashed ZnO_citrate_ film displayed two notable peaks at 1578 and 1387 cm^–1^, corresponding to the antisymmetric and symmetric stretches of the −COO^–^ groups.^21^ Upon adsorption, these peaks shifted significantly by 16 and 37 cm^–1^, respectively, compared to those of pure citrate. In stark contrast, the shifts for the other acids were substantially smaller: 4 and 16 cm^–1^ for glycolate, 10 and 1 cm^–1^ for malate, 1 and 4 cm^–1^ for maleate, and 4 and 6 cm^–1^ for succinate. This confirms that citrate possesses a superior and more specific adsorption affinity for the ZnO surface, a trait also observed in conventional slow-growth methods.

**Figure 11:**
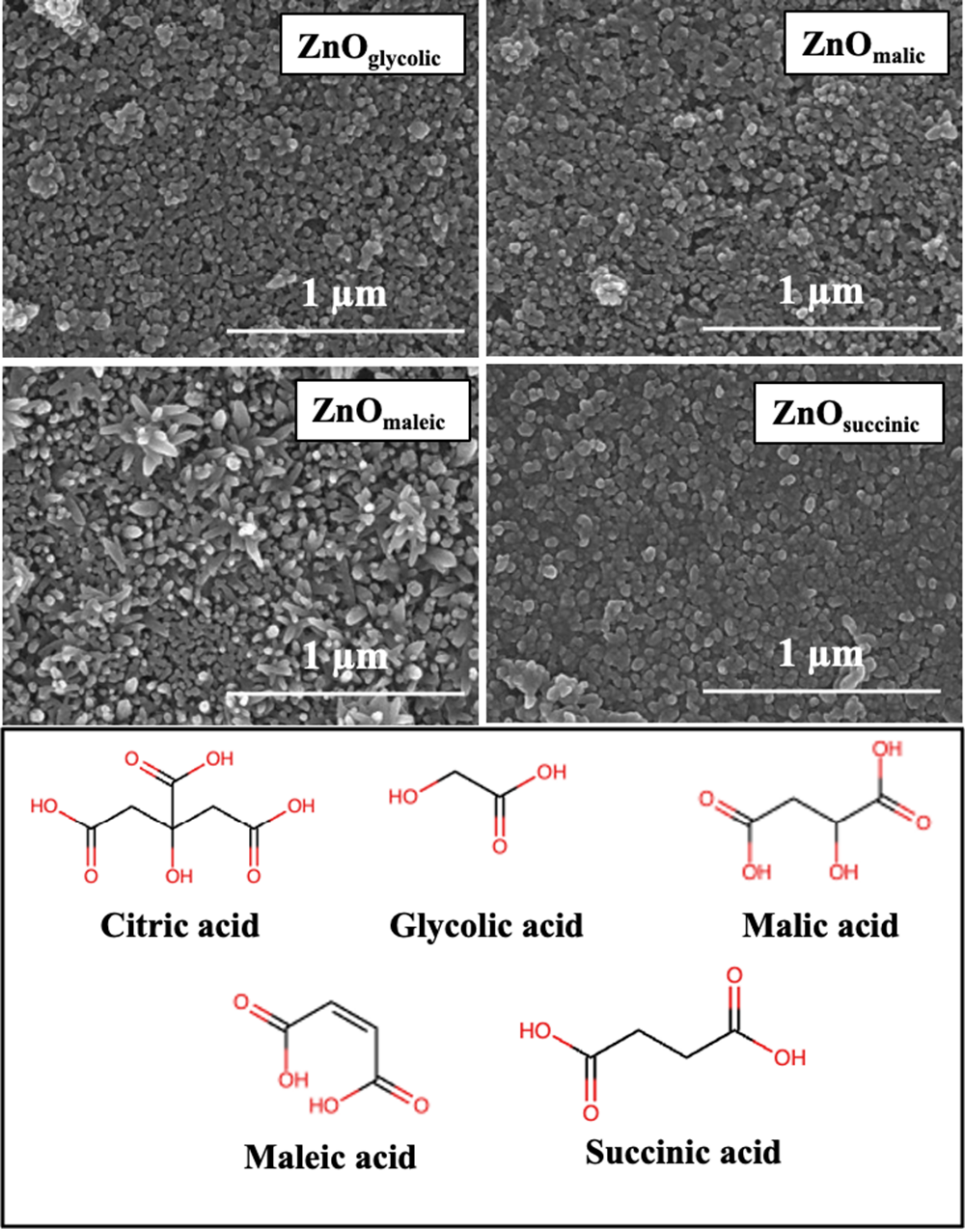
SEM images of ZnO_additives_ thin films (glycolic, malic, maleic, and succinic acid) and their chemical structures.

**Figure 12:**
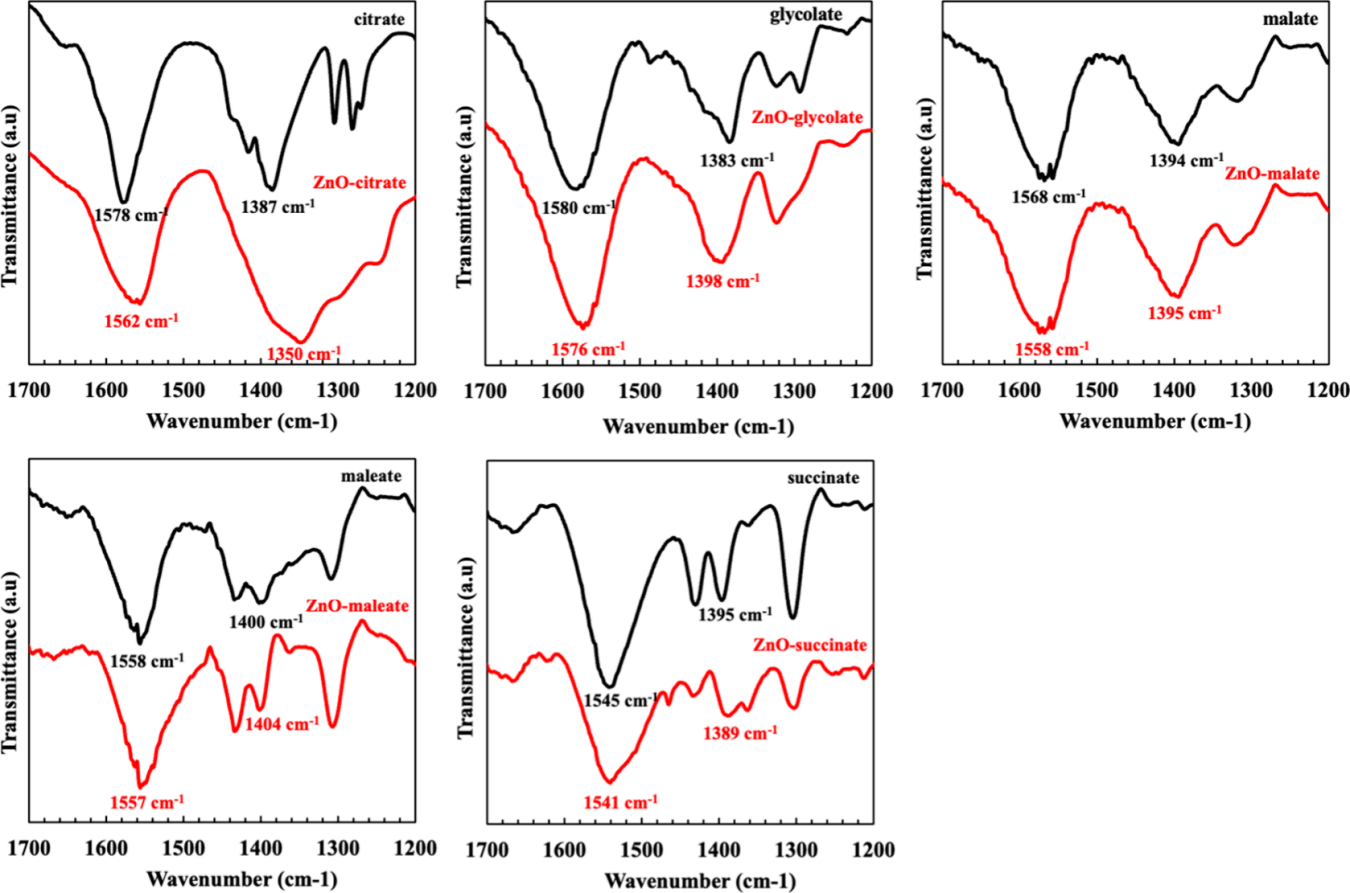
FT-IR spectra of ZnO_additives_ thin films fabricated without cleaning, compared to the additives thin films themself.

This strong adsorption affinity, however, leads to a critical paradox when comparing its behavior across different synthesis regimes. In conventional slow-growth methods where Zn­(OH)_2_ is the growth unit, this strong interaction often results in permanent incorporation of citrate as an impurity, leading to the well-documented suppression of [0001] growth.^6,35^ In stark contrast, within our Zn­(OH)_4_^2–^-driven fast-growth process, the same strong interaction creates a completely transient surface agent that is easily washed away (as evidenced by Figure 1), yet paradoxically acts as a powerful growth promoter. This dichotomy—where strong adsorption leads to either static entrapment and inhibition or a dynamic, transient promotion—provides compelling evidence that the presence of the Zn­(OH)_4_^2–^ growth unit fundamentally alters the nature of the surface interaction. It suggests that for the [0001] promotion to occur, two conditions must be met: the presence of the correct growth unit in solution and a surface modifier with a sufficiently strong and specific binding affinity to effectively mediate the crystallization process.

### 3.3 Proposed Mechanism of Kinetically Controlled Growth Promotion

Based on our collective evidence, we propose a mechanism centered on the Zn­(OH)_4_^2–^ growth unit activating a fundamental change in the nature of the citrate–surface interaction. An alternative hypothesis, based on conventional electrostatic competition and steric hindrance, might suggest that the dominant growth unit competes with citrate for adsorption sites on the (0002) facet.^36^ This model is, in fact, highly effective at explaining the conventional suppressor role of citrate observed in slow-growth methods in which the growth unit is Zn­(OH)_2_. In that regime, as this hypothesis would correctly predict, the neutral Zn­(OH)_2_ growth units cannot effectively compete with the strongly adsorbing anionic citrate. Citrate thus “wins” the competition, successfully passivates the (0002) facet, and inhibits [0001] growth. This competitive framework is also useful to explain the crystal growth behavior of ZnO without additives, as pointed out in our previous work.^18^ The neutral Zn­(OH)_2_ units showed poor affinity for the (0002) surface, and almost no crystal growth occurred, leading to nanoparticles, while the anionic Zn­(OH)_4_^2–^ units have a significantly stronger interaction, promoting nanorod growth. However, this same competitive model, which works well for the slow-growth methods and no-additive cases, cannot account for our key experimental data when citrate is combined with the fast-growth Zn­(OH)_4_^2–^ regime. It fails to explain why citrate is a transient, removable agent in our fast-growth method, confirmed by FT-IR results in Figure 1, rather than a permanently trapped impurity as seen in slow-growth methods. Furthermore, this hypothesis faces a challenge in explaining why citrate acts as a powerful active promoter, confirmed by SEM results in Figure 2, where more citrate leads to enhanced growth. Therefore, we conclude that the Zn­(OH)_4_^2–^ growth unit does not simply displace citrate. Therefore, we discussed these advanced mechanisms explaining how the switch from Zn­(OH)_2_ to Zn­(OH)_4_^2–^ growth unit inverts function of citrate. First, we propose that under fast-kinetic conditions, citrate adsorption behavior transitions from a static inhibitor to a dynamic surface modifier. The diffusion of adsorbed species on a growing crystal surface is a fundamental process in crystal engineering.^37–40^ For large, multidentate molecules like citrate, this can occur via a complex, coordinated “walking” mechanism, where the molecule moves across the facet without fully desorbing.^10^ Critically, such dynamic motion requires a sufficient energetic driving force, which we propose is provided by the high reaction rate of the Zn­(OH)_4_^2–^ growth unit under our fast-kinetic MSS conditions. Conversely, under slow-growth conditions, where Zn­(OH)_2_ is the primary growth unit, this driving force is insufficient, leading to reduced citrate mobility. The molecule then acts as a static inhibitor, leading to the suppression of [0001] growth and potential entrapment within the thin films. Our FT-IR analysis (Figure 1) provides strong support for this model, confirming the absence of residual citrate in the washed MSS-fabricated thin films, in sharp contrast to reports from conventional slow-growth methods, where citrate is often detected as an impurity. However, the surface diffusion mechanism only explains why [0001] growth is not inhibited; it does not fully account for the [0001] growth promotion when citrate was used in MSS method. We therefore propose a second mechanism: the reduction of the crystallization energy barrier. When citrate is introduced, it adsorbs onto the (0002) surface, serving a similar role to native −OH groups in stabilizing the positive charge of ZnO(0002) facet. Crucially, the binding energy of the resulting carboxylate-zinc (−COO–Zn) bonding (0.45–2.45 eV)^11,41,42^ is significantly lower than that of the Zn–OH bond (3.67–4.27 eV).^43,44^ This substitution of stronger Zn–OH bonds with weaker −COO–Zn bonds effectively lowers the energy barrier for the incorporation of Zn­(OH)_4_^2–^ units into the crystal lattice, thereby accelerating the growth rate along the [0001] direction. This mechanism is directly supported by our XPS results, which show the progressive decrease and eventual disappearance of surface −OH groups upon citrate addition.

## 4 Conclusions

In this work, we demonstrate that the function of trisodium citrate in ZnO growth can be fundamentally inverted from a conventional [0001] suppressor to a powerful promoter by shifting the synthesis from a slow- to a fast-kinetic regime. This reversal is attributed to a switch in the dominant growth unit from Zn­(OH)_2_ to the highly reactive Zn­(OH)_4_^2–^ anion. We propose a dual mechanism where citrate acts as a dynamic modifier rather than a static blocker and, more critically, lowers the crystallization energy barrier by replacing surface Zn–OH groups with weaker −COO–Zn linkages, a model strongly supported by our experimental results. This kinetically controlled strategy enables the synthesis of high-aspect-ratio ZnO nanorods with enhanced photocatalytic performance, demonstrating a powerful approach for material design.

## Supplementary Material



## Notes

Detailed schematic of the fabrication apparatus; photographic images of the films; additional SEM images of the effects of deposition time and molar ratios; and supplementary XRD spectra (PDF)
