# Supplementary material for: Functional Inversion of Trisodium Citrate from a [0001] Growth Suppressor to a Promoter in ZnO Thin Film Fabrication
Source: ACS Omega. 2026 Jan 9;11(3):3916–25. doi: 10.1021/acsomega.5c07099 (PMC12854508; doi:10.1021/acsomega.5c07099)
Supplement: Supplementary file 1 [file ao5c07099_si_001.pdf]

Functional Inversion of Trisodium Citrate from a [0001] Growth Suppressor to a Promoter in ZnO Thin Film Fabrication

Lo Tuan Son,[1] Yuta Kubota,[1] Hajime Wagata,[2] Nobuhiro Matsushita[1*]

[1] Department of Materials Science and Engineering, School of Materials and Chemical Technology, Institute of Science Tokyo, 2–12–1 Ookayama, Meguro, Tokyo 152–8550, Japan

[2] Department of Applied Chemistry, School of Science and Technology, Meiji University, 1 Chome-1-1 Higashimita, Tama Ward, Kawasaki, Kanagawa 214-8571, Japan

Supporting information

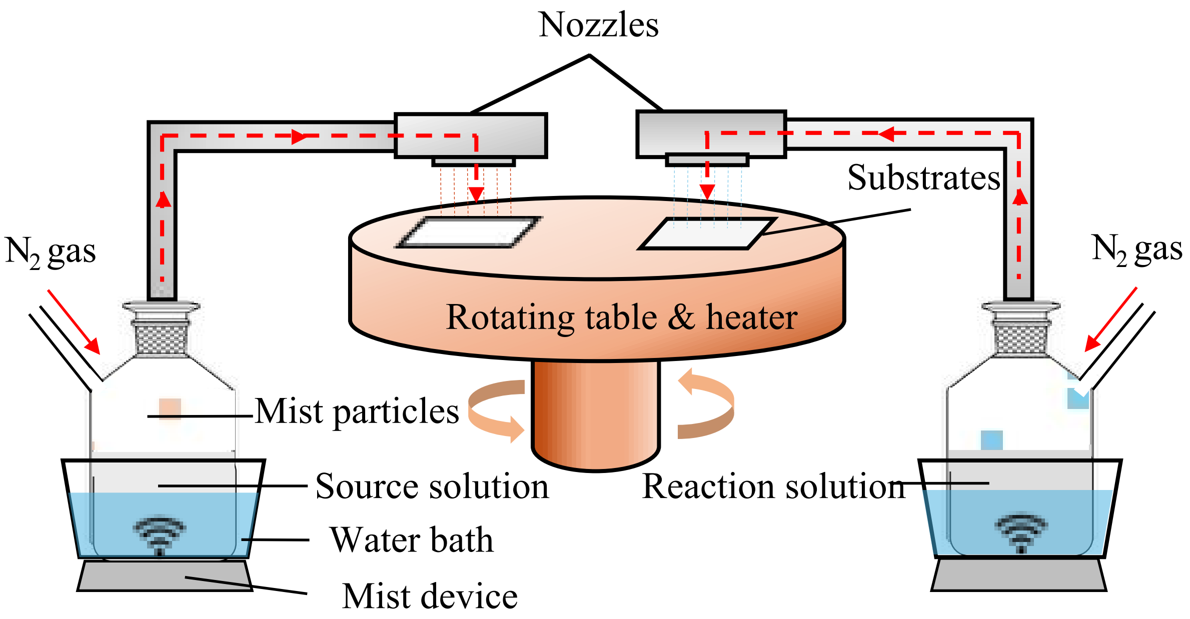

Figure S1. Schematic illustration of the Mist Spin Spray (MSS) fabrication apparatus. The setup consists of two sealed containers, each holding a different precursor—a source solution and a reaction solution. An ultrasonic transducer located at the bottom of each container, atomizes the liquid into a fine mist. A carrier gas is fed into each container, which then transports the aerosolized precursor mist through separate tubes to a corresponding nozzle. The two mists are simultaneously sprayed from their respective nozzles onto substrates. These substrates are mounted on a central platform that functions as both a heater and a rotating table.

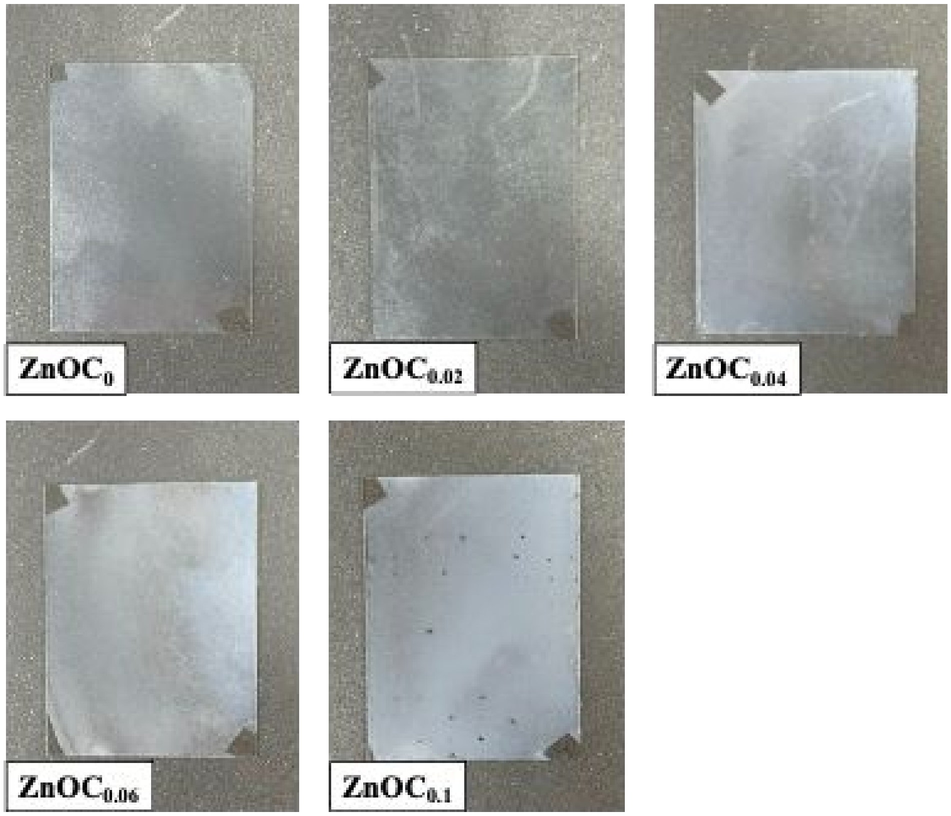

Figure S2. Photographic images of ZnO_{Cx} thin films (x=0-0.1)

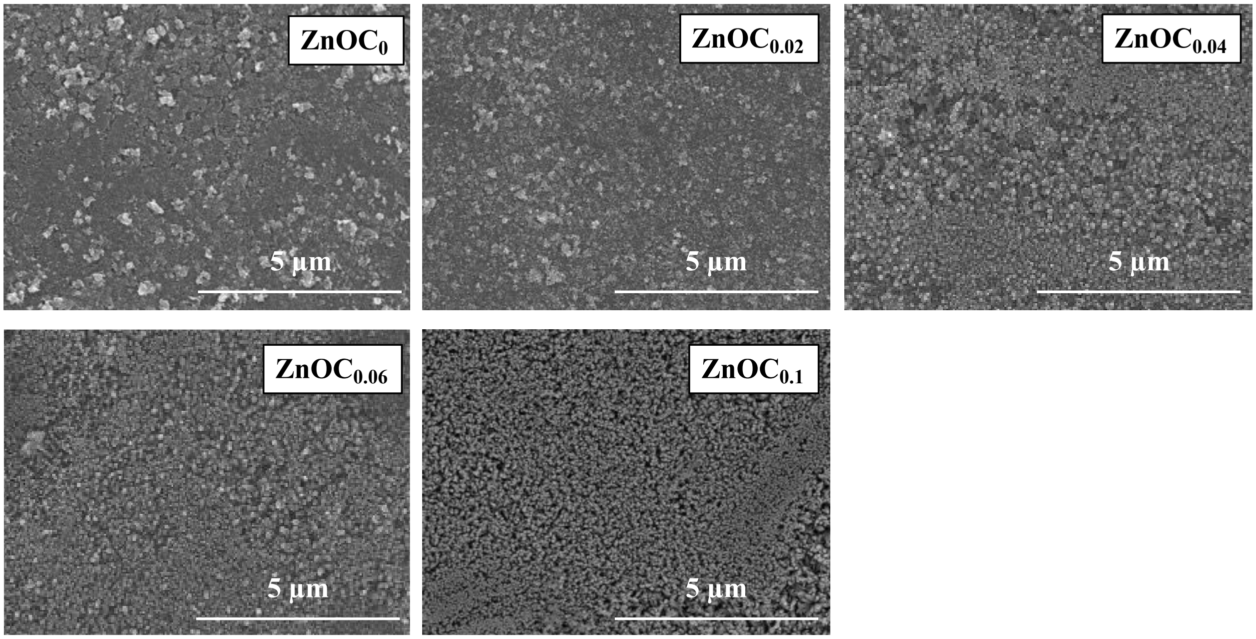

Figure S3. SEM images (10000 times magnification) of ZnO_{Cx} thin films (x=0-0.1)

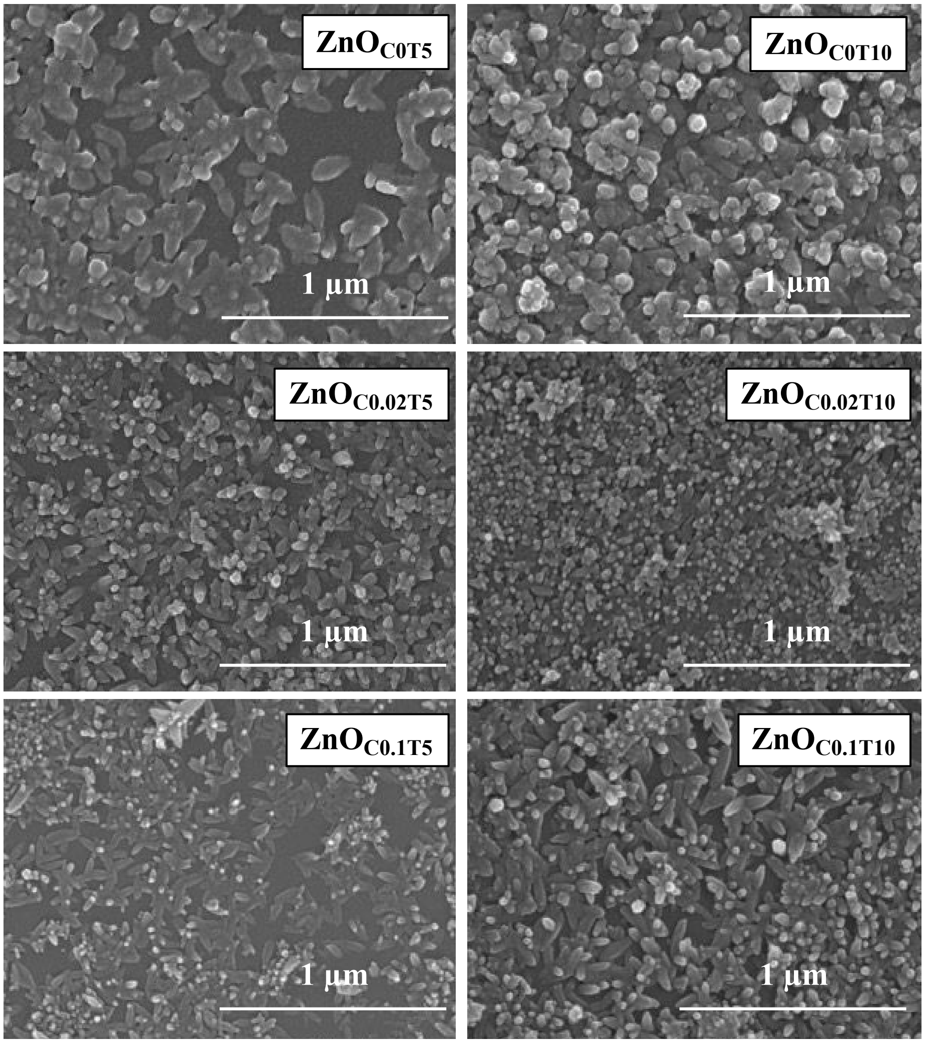

Figure S4. SEM imgages of ZnO$_{CxTy}$ thin films (x=0, 0.02, and 0.1; y = 5, and 10)

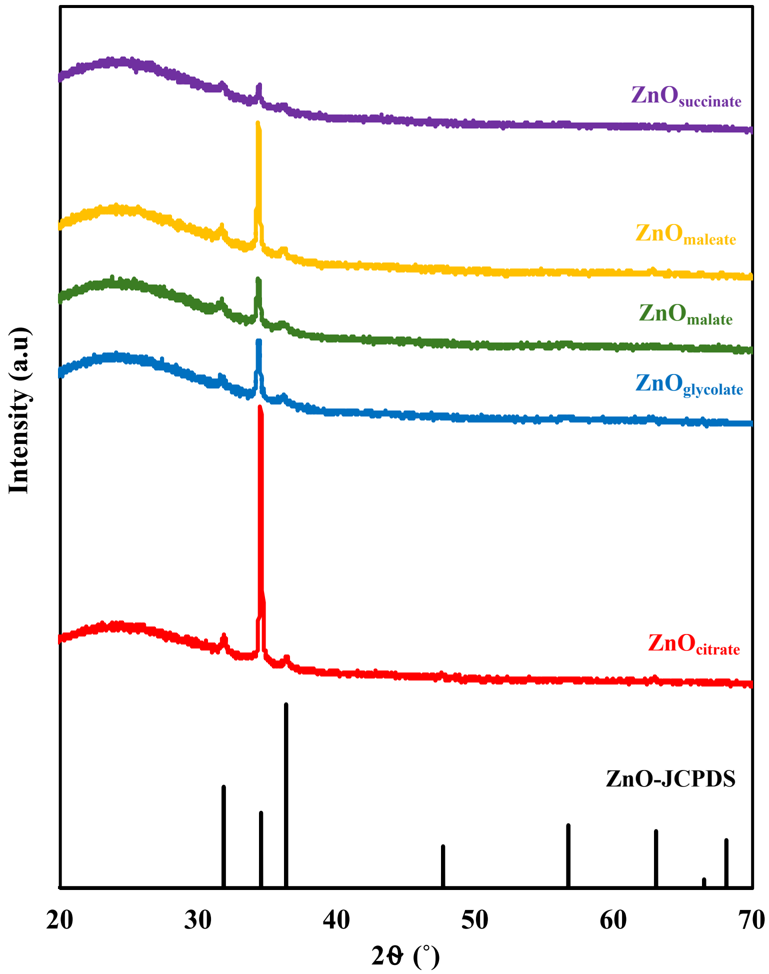

Figure S5. XRD spectra of ZnO$_{citrate}$, ZnO$_{glycolate}$, ZnO$_{malate}$, ZnO$_{maleate}$, and ZnO$_{succinate}$

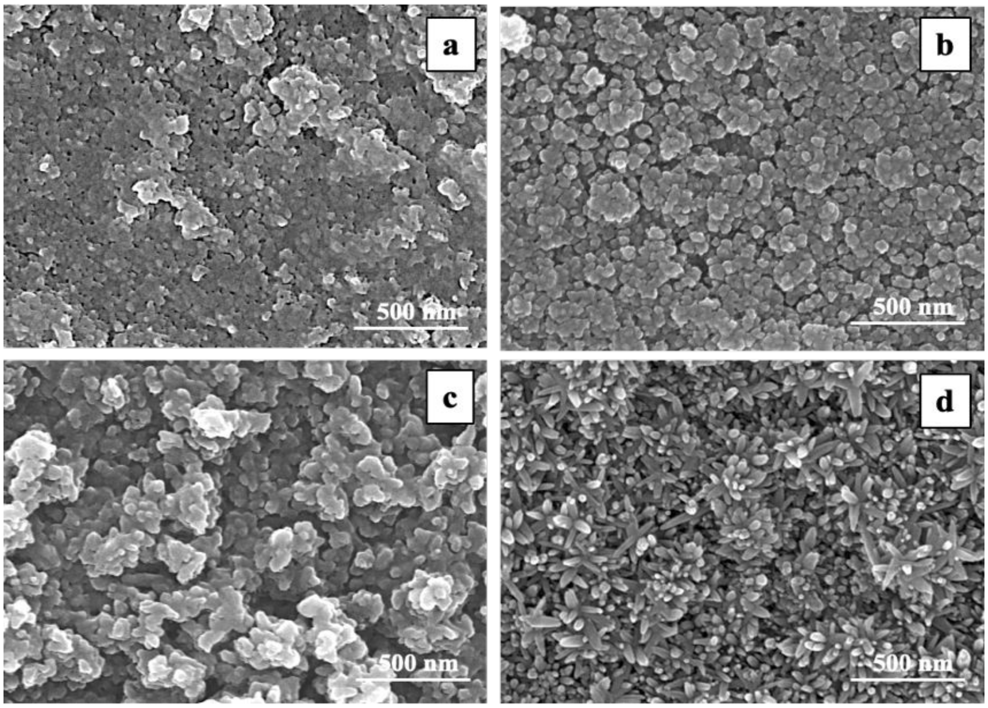

Figure S6. SEM images of: a) $ZnO_{C0R1:2}$, b) $ZnO_{C0.06R1:2}$, c) $ZnO_{C0R1:3}$, and d) $ZnO_{C0.06R1:3}$
